# Efficacy and safety of Zuogui Pill in treating osteoporosis: Study protocol of a systematic review

**DOI:** 10.1097/MD.0000000000013936

**Published:** 2019-02-22

**Authors:** Guoming Chen, Zhaoping Zhang, Yunyun Liu, Jiaxin Lu, Xiangjun Qi, Caishan Fang, Chi Zhou

**Affiliations:** a Guangzhou University of Chinese Medicine; b Department of Orthopedics, First Affiliated Hospital of Guangzhou University of Chinese Medicine, Guangzhou, China.

**Keywords:** osteoporosis, protocol, systematic review, Zuogui Pill

## Abstract

**Background:**

Osteoporosis (OP) is a common skeletal disorder worldwide, resulting in increased bone fragility and high risk of fractures. The Zuogui Pill (ZGP), a classic Chinese herbal formulation, has played a vital role in the clinical practice of OP in China for centuries. Increasing studies have been performed for clarifying its anti-osteoporotic mechanisms. However, this treatment still lacks a systematic review for its efficacy and safety in the treatment of OP.

**Methods:**

Eight electronic databases will be searched from inception to November 2018 by 2 independent researchers, in order to collect qualified randomized controlled trials (RCTs) on the ZGP treatment for OP. The therapeutic effects according to bone mineral density (BMD) will be adopted as the primary outcomes. RevMan V.5.3 software will be used for the data synthesis and the Cochrane's risk of bias assessment tool will be used to assess the risk of bias.

**Results:**

This review will conduct a high-quality synthesis on present evidence of ZGP for OP.

**Conclusion:**

The conclusion of the study will indicate whether ZGP is an effective treatment for OP by providing updated evidence.

**PROSPERO registration number:** PROSPERO CRD 42018114366

## Abstract 2

Supplemental Digital Content is available in the text

## 1 Introduction

### 1.1 Rationale of the condition

Osteoporosis (OP) is a worldwide skeletal disorder characterized by loss of bone mass, microarchitectural deterioration, and decline in bone quality, which causes increased skeletal fragility and high risk of fractures.^[1,2]^ OP can occur at any age, but more often in the elderly and postmenopausal women.^[3]^ As a costly chronic conditions, OP is a major public health crisis with such a high prevalence that 1 in 3 women and 1 in 5 men over the age of 50 will have a bone fracture related to osteoporosis.^[4]^ OP has been usually described as a silent disease for its lack of obvious symptoms until the occurrences of fractures, the most common complication of OP, probably leading to a poor prognosis like disability.^[5]^

The bone-remodeling cycle including osteoclasts and osteoblasts will be out of balance and OP will happen when any of the factors involved in bone remodeling—muscle signals, hormone regulation, bone resorption, or bone formation goes wrong.^[4,6,7]^ These are also the options and targets of the treatment strategies for OP. Currently, pharmacotherapy still remains the main therapeutic method for OP, including hormone replacement therapy (HRT; oestrogen with or without progestin), calcitonin salmon, selective oestrogen receptor modulators (SERMs), bisphosphonates, synthetic parathyroid hormone, mineral agent, and Biologic-RANK ligand inhibitor (Denosumab).^[7–9]^ However, lots of studies have shown that most of them pose problems with long-term use due to their limitations and side effects.^[4,10–16]^ With the aging of population worldwide, the increasing health burden and costs attributed to osteoporotic fractures urge us to explore other helpful therapies.

Chinese herbal medicines and alternative therapies have been used to treat OP for thousands of years in China.^[16–18]^ In traditional Chinese medicine (TCM), the incidence of OP is closely associated with the deficiency of kidney-yin, a crucial substance closely related to the growth and development of bones. Tonifying kidney-yin is therefore considered to be a major therapy for OP in TCM.

### 1.2 Rationale of the intervention

Zuogui Pill (ZGP), a famous Chinese herbal formulation for tonifying kidney, has played an essential role in the clinical practice of OP in China for centuries. The herbs of the formula include Rehmanniae Radix Praeparata (*shu di huang*), Dioscoreae Rhizoma (*shan yao*), Lycii Fructus (*gou qi zi*), Corni Fructus (*shan zhu yu*), Cyathulae Radix (*chuan niu xi*), Cuscutae Semen (*tu si zi*), Cervi Cornus Colla (*lu jiao jiao*), and Testudinis Carapacis Et Plastri Colla (*gui jia jiao*). Currently, more and more efforts have been made to expound the anti-osteoporotic effects and mechanisms of ZGW. These studies found that ZGP has positive effects in reversing the imbalance of bone formation and bone resorption through multiple targets and pathways, including upregulation of MAPK, Wnt/*β*-catenin, and TGF-*β*1/Smad signaling pathways.^[19–25]^ However, there is still remaining no systematic review to present evidence of effectiveness and safety of ZGP for OP.

### 1.3 Objectives

Consequently, our study is performed to provide a systematic review in terms of the efficacy and safety of ZGP in the treatment of OP, based on the collection and analysis of related clinical randomized controlled trials (RCTs).

## 2 Methods

### 2.1 Inclusion criteria for study selection

#### 2.1.1 Types of studies

All RCTs of ZGP as the sole treatment for OP will be included, regardless of their language and publication status. In addition, studies like Quasi RCTs, cohort, case reports, comments, case series, reviews, and animal experiments will be excluded.

#### 2.1.2 Types of patients

Regardless of race, region, sex, age, severity or duration, patients clinically diagnosed with OP in accordance with the standard established by the UK National Osteoporosis Guideline Group (NOGG) or Expert consensus on diagnostic criteria for Chinese osteoporosis will be considered eligible for the study. Patients with other types of diseases like diabetes, hyperparathyroidism, ankylosing spondylitis, and other diseases that may affect bone metabolism will be ruled out.

#### 2.1.3 Types of interventions

Patients in the treatment group will only receive a prescription including the main ingredients of ZGP based on traditional East Asian medicine theories, while the control group using no treatment or placebo. Without limit on formulations, decoction, tablets, pills, powder, and any other types of ZGP will be accepted in the study.

#### 2.1.4 Types of outcome measures

##### 2.1.4.1 Primary outcomes

Bone mineral density (BMD).

##### 2.1.4.2 Secondary outcomes

Hip-structure analysis (HSA) variables, bone turnover markers, serum cholesterol concentration, triglyceride concentration, height, physical function, and adverse events, etc.

### 2.2 Search methods for the identification of studies

#### 2.2.1 Electronic searches

Eligible RCTs will be comprehensively collected through searching the following databases from inception to the present date (November 20, 2018): PubMed, EMBASE, Cochrane Library, Web of Science, Chinese Biomedical Database (CBM), China National Knowledge Infrastructure (CNKI), Wanfang database and Technology Periodical database (VIP). Searched terms like ZGP, OP, and RCT will be covered. To decide the final retrieval strategy, several pre-retrievals will be conducted by combining primary keywords and adjustable terms. The information of the final search strategy for PubMed will be presented in Supplement 1, which will be modified when completing other searches.

#### 2.2.2 Other searches

To avoid omission, our researchers will also manually retrieve relevant conference papers, references lists of included studies and gray literature.

### 2.3 Data collection and analysis

#### 2.3.1 Selection of studies

**Figure 1:**
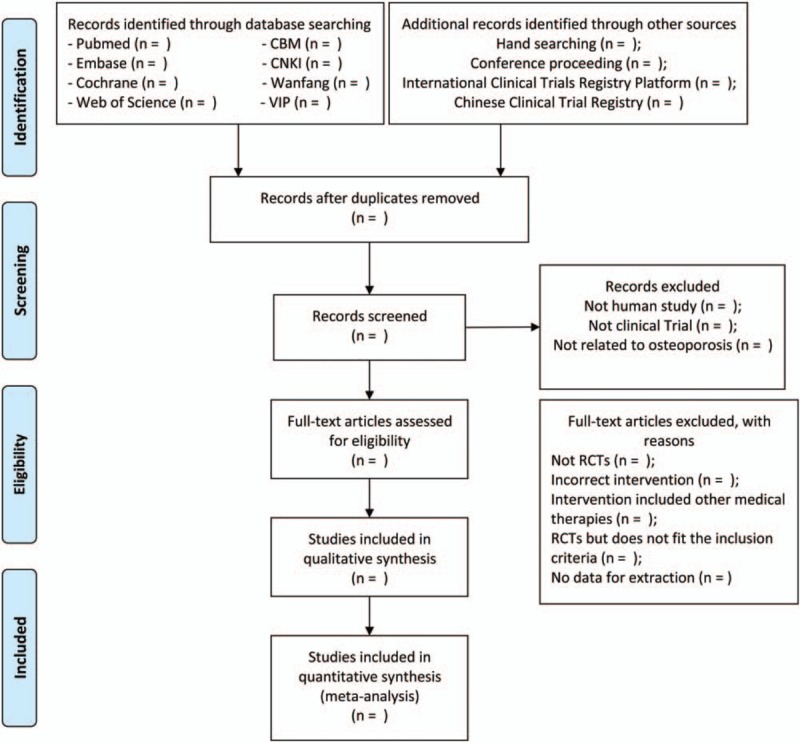
PRISMA flow chart of study selection process. PRISMA = Preferred Reporting Items for Systematic Reviews and Meta-Analyses.

The literature will be imported into the literature management system of EndnoteX9 and the duplicates will be removed. Two researchers will separately conduct the study selection and cross-check. Following the exclusion of the obvious unqualified articles by screening the title and abstract, the rest of the full text will be carefully reviewed in accordance with the inclusion criteria created beforehand. In case of discrepancies, a third researcher will join the discussion and solve the inconsistencies. The final included studies will be documented and summarized. The whole process is presented by Preferred Reporting Items for Systematic Reviews and Meta-Analysis (PRISMA) flow diagram (Fig. 1).

#### 2.3.2 Data extraction and management

Two researchers will extract the basic data and outcome data from the included studies according to the predefined data extraction form, including but not limited to the included trials, first author, publication time, sample size, course and severity of disease, age, sex, disease duration, detailed intervention, outcome indicators, adverse events, and risk of bias assessment. Any disagreements will be arbitrated by the third reviewer after discussion and the final extraction will be crosschecked.

#### 2.3.3 Dealing with the missing data

Where there are ambiguities or missing data, the first author or the corresponding author will be contacted to solve the problem. The potential impact of insufficient information will also be considered in the Discussion Section.

#### 2.3.4 Assessment of risk of bias in included studies

Basing on Cochrane Handbook for Systematic Reviews of Interventions, 2 independent research members will appraise the risk of bias in adopted studies. The results will be shown by utilizing scores of “L,” “U,” and “H,” which respectively indicates a low risk, an uncertain risk and a high risk of bias. Seven domains will be evaluated, namely random sequence generation, allocation concealment, blinding of participants and personnel, blinding of outcome assessment, completeness of outcome data, selective reporting, and other sources of bias. Where there is divergence, the problem will be coped with by discussion and arbitration with a third researcher, and the author will be contacted for confirming details when necessary.

#### 2.3.5 Measures of treatment effect

Mean difference (MD) with 95% confidence interval (CI) will be adopted to indicate the treatment effect for continuous data, while relative risk (RR) will be adopted for dichotomous data with 95% CI.

#### 2.3.6 Assessment of heterogeneity

The heterogeneity will be analyzed by employing *I*^2^ statistic value. A fixed effect model will be used if there is no evidence of substantial heterogeneity (*I*^2^ <50%), otherwise a random model will be applied.

#### 2.3.7 Assessment of reporting bias

If the number of included studies is >10, the publication bias will be visually detected by funnel plots. Begg and Egger test will be performed when asymmetry is presented, and *P* > .05 will suggest no significant publication bias. Taking that funnel plot asymmetry is not equal to publication bias, the possible reasons for it will be distinguished, like poor methodological quality, small study size effects, true heterogeneity, and so forth.

#### 2.3.8 Data synthesis

The systematic review will be completed via using RevMan 5.3. As it is mentioned above, MD or RR will be computed with fixed or random model with 95% CI taking account of the heterogeneity. When apparent heterogeneity is shown, the sensitivity or subgroup analysis will be utilized to explore the source of it. The evidence will be described and summarized only when it comes to the situation that the data are insufficient for quantitative analysis.

#### 2.3.9 Sensitivity analysis

Sensitivity analysis will be generated by ruling out the low-quality studies or small-sample-size studies and reconducting the meta-analysis, and thus exploring whether these factors affect the results. Only when the included studies is enough (>10 RCTs) can the sensitivity analysis be performed.

#### 2.3.10 Subgroup analysis

Aiming at finding out the heterogeneity resources, subgroup analysis will be accomplished by the various characteristics of the studies. Characteristics like OP course, type of intervention, dosage, publication time, race and other aspects will be taken into account for subgroups.

#### 2.3.11 Quality of evidence

To evaluate the quality of evidence of the study, the Grading of Recommendations Assessment, Development and Evaluation (GRADE) will be used. The summary of findings table will be completed to present the result.

#### 2.3.12 Ethics and dissemination

Since the protocol is for a systematic review and no privacy data will be involved, ethical approval and informed consent are not necessary in this study. The findings of the study will be submitted to peer-reviewed publications and conference presentations, so as to be disseminated as widely as possible.

## 3 Discussion

OP belongs to the category of “bone impotence” in traditional Chinese medicine. It is acknowledged in TCM that the kidneys contain an “essence” that generate marrow and regulate bone metabolism. Kidney deficiency is therefore considered to be an important cause of OP. ZGP was first described in Complete Works of Jingyue (*Jing yue quan shu*) in the Ming Dynasty (AD 1368–1644). It is a major kidney-tonifying medication which can effectively improve OP symptoms.

Among the ingredients of ZGP, the chief herb Rehmanniae Radix Praeparata (*shu di huang*) is good at enriching the true yin of the Kidney. Corni Fructus (*shan zhu yu*), Lycii Fructus (*gou qi zi*), and Dioscoreae Rhizoma (*shan yao*) have the effects on tonifying the yin of liver, kidney, and spleen. Testudinis Carapacis Et Plastri Colla (*gui jia jiao*) can invigorating Yin, while Cervi Cornus Colla (*lu jiao jiao*) can tonify Yang. The above 5 are ministerial drug. The assistants, Cuscutae Semen (*tu si zi*) and Cyathulae Radix (*chuan niu xi*), strengthen the bones and muscles. The combination of these herbs plays the role of nourishing the liver and kidney and filling up the marrow, so that it's specialized in treating the symptoms of kidney-yin deficiency in OP.

The increasing pharmacological research has suggested that ZGP has positive effects on treating OP, which mainly reflects in reversing the imbalance between formation and resorption of bone through multiple targets and pathways, including OPG/RANK/RANKL shaft, Wnt/β-catenin signaling pathway, Notch signaling pathway, cAMP/PKA/CREB signaling pathway, MAPK signaling pathway, and TGF-β1/Smad signaling pathway.^[26–28]^

However, a systematic review of ZGP in treating the symptoms of OP has not yet been published. This systematic review will provide a summary of the current state of evidence respecting the efficacy and safety of ZGP in treating the symptoms of kidney-yin deficiency in OP.

## Author contributions

Authorship: Chi Zhou is the guarantor of the article and will be the arbitrator when meeting disagreements. All research members participated in developing the criteria and drafting the protocol for this systematic review. Jiaxin Lu and Xiangjun Qi established the search strategy and they will obtain the hard copies of all articles. Guoming Chen and Zhaoping Zhang will independently accomplish the study selection and data extration and assess the risk of bias. Guoming Chen, Zhaoping Zhang, and Yunyun Liu will perform the data syntheses. The subsequent and final versions of the protocol are critically reviewed, modified and authorized by all authors.

**Conceptualization:** Guoming Chen, Xiangjun Qi.

**Data curation:** Zhaoping Zhang, Yunyun Liu, Jiaxin Lu.

**Investigation:** Zhaoping Zhang.

**Methodology:** Guoming Chen, Yunyun Liu.

**Project administration:** Guoming Chen, Chi Zhou.

**Resources:** Guoming Chen, Chi Zhou.

**Supervision:** Guoming Chen.

**Writing – original draft:** Guoming Chen, Zhaoping Zhang, Yunyun Liu, Jiaxin Lu, Xiangjun Qi, Caishan Fang.

**Writing – review & editing:** Guoming Chen, Chi Zhou.

Chi Zhou orcid: 0000-0003-1905-9494.

## Supplementary Material

Supplemental Digital Content
