# Supplementary material for: Efficacy and safety of Zuogui Pill in treating osteoporosis: Study protocol of a systematic review
Source: Medicine (Baltimore). 2019 Feb 22;98(8):e13936. doi: 10.1097/MD.0000000000013936 (PMC6407973; doi:10.1097/MD.0000000000013936)
Supplement: Supplemental Digital Content [file medi-98-e13936-s001.doc]

**Supplement 1. Search strategy used in PubMed database**

#1 Osteoporoses OR Osteoporosis, Post-Traumatic OR Osteoporosis, Post Traumatic OR Post-Traumatic Osteoporoses OR Post-Traumatic Osteoporosis OR Osteoporosis, Senile OR Osteoporoses, Senile OR Senile Osteoporoses OR Osteoporosis, Involutional OR Senile Osteoporosis OR Osteoporosis, Age-Related OR Osteoporosis, Age Related OR Bone Loss, Age-Related OR Age-Related Bone Loss OR Age-Related Bone Losses OR Bone Loss, Age Related OR Bone Losses, Age-Related OR Age-Related Osteoporosis OR Age Related Osteoporosis OR Age-Related Osteoporoses OR Osteoporoses, Age-Related

#2 Zuogui Pill OR [Zuoguipill OR Zuogui Wan OR Zuoguiwan OR Zuo gui OR zuo-gui OR Zuogui](https://www.ncbi.nlm.nih.gov/pubmed/27473956)

#3 Randomized controlled trial OR clinical study OR Clin-ical Trial OR Controlled study OR Controlled Trial OR Random*Control* study OR random* Control* Trial

#1 AND #2 AND #3
